# Clinical Characteristics of Actinic Keratosis Associated with the Risk of Progression to Invasive Squamous Cell Carcinoma: A Systematic Review

**DOI:** 10.3390/jcm11195899

**Published:** 2022-10-06

**Authors:** Alise Balcere, Laura Konrāde-Jilmaza, Laura Agnese Pauliņa, Ingrīda Čēma, Angelika Krūmiņa

**Affiliations:** 1 Department of Dermatology and Venereology, Riga Stradiņš University, LV-1010 Riga, Latvia; 2 Residency Development Program, University of Latvia, LV-1050 Riga, Latvia; 3 Faculty of Medicine, Riga Stradiņš University, LV-1007 Riga, Latvia; 4 Department of Oral and Maxillofacial Surgery and Oral Medicine, Riga Stradiņš University, LV-1007 Riga, Latvia; 5 Department of Infectiology, Riga Stradiņš University, LV-1006 Riga, Latvia

**Keywords:** AK patch, merging AK, baseline AK, keratinocyte cancer, progression

## Abstract

Background: Actinic keratosis (AK) is one of the most common lesions on chronically sun-damaged skin that has the risk of progression to invasive squamous cell carcinoma (SCC). With the possibilities of using digital technologies for following-up skin lesions and their increased use in the past few decades, our objective was to update the review by Quaedvlieg et al., 2006, and to review prospective studies from 2005 onwards to identify the clinical characteristics of AK that later progressed to SCC. Methods: The PubMed, Scopus, and ScienceDirect databases were searched for relevant articles. The search had the following criteria: English language, human subjects and year from 2005 onwards. The study protocol was registered in the Prospero database with the record number CRD42020200429 and followed the PRISMA guidelines. The risk-of-bias assessment was performed using the QUIPS tool. Results: From the 5361 studies screened, 105 reports were evaluated for eligibility, and 2 articles with 621 patients were included. The main AK types associated with the development of SCC were found to be baseline AK, also known as a long-standing AK, and merging AK, also called an “AK patch”.

## 1. Introduction

Squamous cell carcinoma (SCC) is the second most common skin cancer in humans [1] and has several distinct in situ and invasive types. Actinic keratosis (AK) is a well-recognized precursor of cutaneous SCC that is caused by the long-term exposure of the skin to ultraviolet radiation [2] and is one of the most common reasons for dermatology office visits [3]. The usual clinical presentation of AK is a scaly erythematous macule or patch on a sun-exposed area. In ambiguous cases, histopathological confirmation is needed to differentiate AK from SCC. The progression from AK to invasive SCC has been previously described [4,5,6], and the risk of progression is estimated to be between 0.025% and 20% for each individual lesion [2,6,7]. A study has shown that the process of progression takes approximately 2 years for lesions that warrant histological confirmation [6].

Although some authors state that it is not possible to predict which AKs will progress to invasive SCC [8] and that these lesions are more a general marker of the risk of SCC than true precursors [9], a study by Quaedvlieg et al., published in 2006 identified several clinical findings associated with the malignant progression of AK and invented the acronym IDRBEU. In the acronym, “I” stands for inflammation/induration; “D”, for a diameter > 1 cm; “R”, for rapid enlargement; “B”, for bleeding; “E”, for erythema; and “U”, for ulceration. Additional minor clinical criteria identified in their study were pain, palpability, hyperkeratosis, pruritus, and pigmentation [10].

As new studies and new technologies have been implemented in dermatology to perform long-term follow-ups of separate lesions, we believe that new evidence of the clinical features of AK progressing to SCC should be available. Therefore, we decided to update the review by Quaedvlieg et al. [10].

## 2. Materials and Methods

The study’s protocol was registered in the Prospero database with the record number CRD42020200429 [11] and followed the PRISMA guidelines for reporting systematic reviews [12].

The PubMed, Scopus, and ScienceDirect databases were searched for relevant studies on 28 July 2020. The search was restricted to years from 2005 onward. The search was limited to papers written in the English language and studies involving human subjects. The following inclusion criteria were used: patients with diagnoses of AK (P), information on follow-up was provided or a longitudinal assessment was performed (I), and the development of SCC (O) was recorded in a prospective manner (S). The exclusion criteria were a lack of data on previous AK (P); no clinical characteristics of AK being mentioned (P); treated AK (I); no prospective data being available (I); no development of SCC being mentioned (O); no data regarding SCC development from AK being presented (O); review articles (S); and articles with follow-up periods less than 3 months (S). The search strings were composed in collaboration with the Riga Stradiņš University Library and can be found in the registered protocol [11]. The search results were extracted and uploaded to the Covidence system for the removal of duplicates and the selection of relevant titles and abstracts. There were minor author changes from the registered protocol. Two authors (A.B. and L.K.Y.) were assigned, and they independently performed the title and abstract selection. In the case that an abstract was unavailable, the full text was screened at the initial stage. All the discrepancies were resolved in discussions with I.C. after the manual extraction of conflicting articles. The full texts of the selected articles were reviewed independently by A.B. and L.A.P.; all the discrepancies were resolved in discussions with L.K.Y. and I.C. The reference lists of eligible studies were manually screened for additional relevant articles. The search was rerun from 2 to 3 February 2022 to include the latest articles. The risk-of-bias assessment for the selected articles was performed using the QUIPS tool for assessing the risk of bias in prognostic factor studies by A.B. and L.K.Y. All the disagreements were resolved in discussions with I.C.

## 3. Results

**Figure 1:**
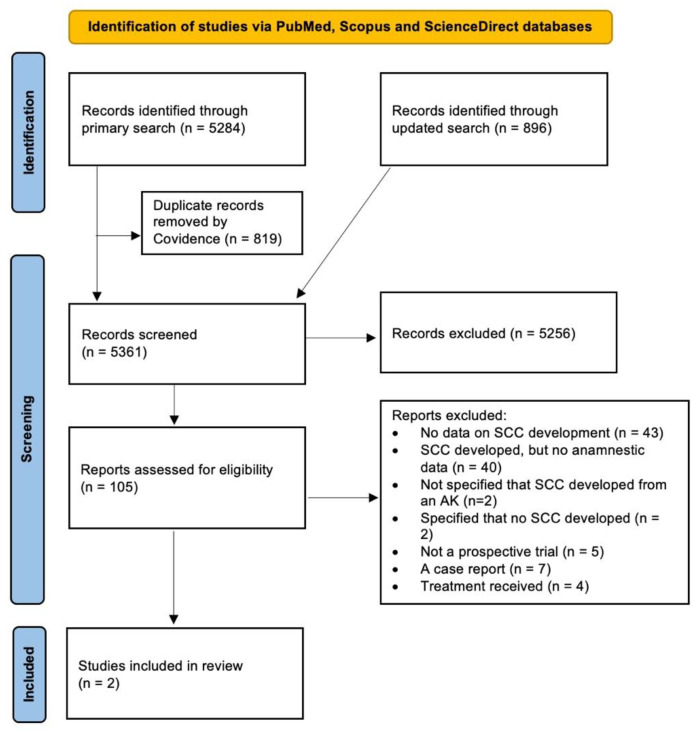
PRISMA flow diagram of the article selection process. Two studies met the inclusion criteria. AK—actinic keratosis. SCC—squamous cell carcinoma.

**Figure 2:**
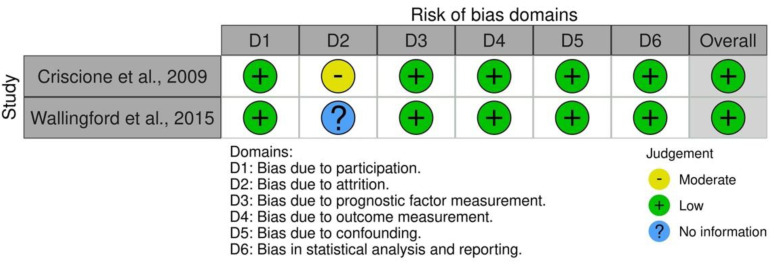
Evaluation of risk of bias using the QUIPS tool. Both studies were considered to have an overall low risk of bias. QUIPS, Quality in Prognosis Studies [13,14].

The PRISMA flow diagram of the article selection process is depicted in Figure 1. In total, 5361 titles and abstracts were screened, 105 articles were evaluated for eligibility, and 2 studies with 621 patients were included [13,14]. Both of the included studies had a low risk of bias, as evaluated with the QUIPS tool (Figure 2) for assessing the risk of bias in prognostic factor studies. Both of the studies had patients at high risk for the development of SCC.

First, a study by Criscione et al. [13] longitudinally examined a group of veterans from the Department of Veterans Affairs Topical Tretinoin Chemoprevention Trial. The study cohort consisted of 169 participants who had been diagnosed with more than two keratinocyte carcinomas in the 5 years prior to enrollment. The participants were examined at approximately 6-month intervals for a mean of 7 examinations (range: 2–16 examinations). During each examination, high-resolution standardized digital photographs were taken. In total, 187 primary SCCs on the face or ears developed in the study. Of those, 65% (91 invasive and 31 in situ SCCs) were diagnosed from previously documented AK. The main type of AK associated with the development of SCC was baseline AK, which showed an increased risk of progression to primary SCC (invasive or in situ; *p* = 0.02) but not to primary invasive SCC (*p* = 0.17). The risk of progression from baseline AK to in situ or invasive SCC was 3.13% at 3 years and 4.03% at 5 years.

In the second study by Wallingford et al. [14], a dermatologist examined and performed a routine follow-up from May 2010 to October 2011 for a representative cohort of 452 white renal transplant recipients (RTRs). In total, 130 (29%) of the participants had AK at the time of examination and were examined by a dermatologist every 3–4 months [15]. The authors defined merging AK, namely, skin areas greater than 1 cm^2^ with confluent erythema and scaling, as actinic field changes. During the study period, 20 (4%) RTRs were diagnosed with SCC. Of those, 11 (55%) developed the malignancy directly in an area of field change. One RTR developed two SCCs in areas of field change at two different sites. The field change increased the risk of SCC by 93-fold.

In both of the included studies, the patients received no specific AK treatment except sunscreen and tretinoin, which had no effect on SCC or AK reduction.

## 4. Discussion

This systematic review identified baseline or pre-existing AKs and large AKs that exceed 1 cm^2^ in diameter as the main clinical features of AK that are associated with the development of SCC in prospective longitudinal studies.

The development of SCCs from baseline or pre-existing AKs is in agreement with the well-established mode of progression and has been supported by clinical and histological studies [5,6]. Clinically, this can be translated as meaning that the lesions initially diagnosed in a patient are more likely to develop into SCC than are those that develop during a follow-up. Similarly, this is in accordance with the time required for the development of SCC: the longer the lesion is present, the higher the probability of SCC development [13].

The development of SCCs from areas of confluent AKs or large AKs that exceed 1 cm^2^ in area agrees with the findings from a previous study by Quaedvlieg et al. [10], who highlighted a diameter of more than 1 cm as a risk factor for the development of SCC. In some studies, large AKs exceeding 1 cm^2^ have been called AK patches and have similarly been associated with the development of SCC. For example, in two studies by Jiyad et al. [16,17], signs predicting the development of SCC in a certain area were AK patches, the number of AK patches, three or more AKs at a single anatomical site, and the percentage of the area involving AK > 25%. Jiyad et al., defined an area by dividing the face into five areas, in addition to each ear as a separate area. Both studies were excluded from our review, as there was no information indicating that SCCs had developed from the AK patches themselves. Furthermore, large areas of confluent AKs have also been linked to the more aggressive behavior of SCCs [18].

Both the included studies had patients at high risk for SCC development, namely, patients with previous keratinocyte carcinomas and renal transplant recipients. It is known that immunosuppressed patients have a higher prevalence of AK and a higher risk of the progression of these lesions to SCC. This among other reasons can be attributed to the use of immunosuppressive medications that cause direct damage to the DNA when the patient is exposed to UVA radiation; in addition, these medications are photosensitizing and also affect the correction pathways of pre-oncogenic mutations [19].

In the previous study by Quaedvlieg et al. [10], the identified clinical features were derived from four literature reviews and a single prospective study by Suchniak et al. [20]. In the latter, it was found that clinical hyperkeratotic AKs less than 1 cm in diameter on the hands, wrists, and forearms of white patients who have had severe actinic damage are often invasive SCCs (in 36% of cases). Therefore, the only clinical feature included in the risk factors associated with a malignant progression of AK into SCC from prospective trials in the study by Quaedvlieg et al. [10] was hyperkeratosis. Our study did not find hyperkeratosis as a feature associated with SCC development in prospective longitudinal studies. Moreover, studies have shown that only 14% of hyperkeratotic lesions correspond to grade III AKs on histopathology with atypical keratinocytes extending to more than two-thirds of the full thickness of the epidermis [21].

In the final step of the screening, five case reports were reviewed, from which additional information was obtained. In these reports, the mean age of the patients was 84 years (range: 73–101), and in all cases, the SCC developed from an AK on the face. In two cases where information on the size of the AK was available before the development of SCC, it was at least 1.6 cm in diameter [22,23]. In four cases, pre-existing AK was present for several years [23,24,25,26], and in four cases, a recent rapid growth was observed [22,24,25,26]. In only one of the reported cases, where SCC developed from a previous AK, was the patient specified to have no other AK lesions [24].

The strengths of this study include the comprehensive data search, the registered protocol, the adherence to reporting guidelines, and the large amount of literature reviewed.

However, there are several limitations of this study. First, only a low number of studies were included. Second, we were unable to identify studies on low-risk patients. The probable reasons include a lower risk of SCC development in immunocompetent patients, complicated study designs, the high costs of such studies, the high numbers of AKs a single individual can have, and the fact that digital follow-up is more commonly used for melanocytic skin lesions. In addition, only databases, but no other sources, were searched.

## 5. Conclusions

In comparison to previous reviews, longitudinally assessed features of AK progression to invasive SCC have been summarized in this article. We conclude that long-standing and large or merging actinic keratoses, sometimes called AK patches, are the most important risk factors for the development of SCC in high-risk populations.

## Data Availability

The study protocol and search string were registered in the Prospero database with the record number CRD42020200429 and can be found at: https://www.crd.york.ac.uk/prospero/display_record.php?RecordID=200429, 20 September 2022.
